# Menstrual suppression to decrease intrauterine device expulsion in adolescents with inherited bleeding disorders

**DOI:** 10.1002/ijgo.16063

**Published:** 2024-11-25

**Authors:** Peter H. Cygan, Kelly M. Kons, Megan H. Fiorillo, Tonya S. Wright

**Affiliations:** ^1^ Division of Blood and Vascular Disorders, Department of Medicine Penn State Health Milton S. Hershey Medical Center Hershey Pennsylvania USA; ^2^ Department of Obstetrics and Gynecology University of Illinois at Chicago Chicago Illinois USA; ^3^ Division of Academic Specialists in Ob/Gyn, Department of Obstetrics and Gynecology Penn State Health Milton S. Hershey Medical Center Hershey Pennsylvania USA

**Keywords:** abnormal uterine bleeding, adolescent gynecology, heavy menstrual bleeding, inherited bleeding disorders, intrauterine device, menstrual suppression

## Abstract

Intrauterine device efficacy in adolescents with heavy menstrual bleeding and bleeding disorders is impacted by expulsion within 30 days of insertion. Menstrual suppression may reduce this risk.

## 1 INTRODUCTION

One in four adolescents with heavy menstrual bleeding (HMB) since menarche may have an inherited bleeding disorder (IBD).^1^ Levonorgestrel‐containing intrauterine devices (LNG‐IUDs) are the preferred treatment for HMB in adolescents owing to the substantial and extended reduction in bleeding and favorable side effect profile.^2^ Medical management alone is frequently insufficient in persons with IBDs,^3^ yet several barriers impact the use of LNG‐IUDs in those with IBDs, including a lack of standardized periprocedural guidelines, perceived higher bleeding risk associated with IUD insertion complications, and placement‐related pain. In addition, a recent history of HMB increases the risk of IUD expulsion threefold.^4^ Moreover, first spontaneous IUD expulsion is more frequent among adolescents, potentially because of small uterine size.^5^, ^6^ Expulsion risk factors include young age, history of anemia, concurrent bleeding disorder diagnosis, and abnormal uterine bleeding.^5^ While the LNG‐IUD is as effective for HMB treatment in adolescents with IBDs as those without IBDs,^7^ a key barrier to securing this extended efficacy is a more frequent IUD expulsion rate of 8%–9% within 30 days of insertion.^2^, ^6^, ^7^ Importantly, expulsions after 30 days are not more frequent than in adolescents without IBDs.^2^

We hypothesize that prophylactic menstrual suppression after IUD insertion may reduce early (≤30 days) device expulsion. Our primary objective was to examine the rates of early IUD expulsion in adolescents with IBDs with concurrent menstrual suppression.

## 2 MATERIALS AND METHODS

The Penn State Health Women and Girls Bleeding Disorder Clinic provides women's health services for patients with suspected or confirmed IBDs, including Ehlers‐Danlos syndrome.^7^, ^8^ This retrospective study was approved by Penn State University's institutional review board and includes adolescent girls (aged 10–21 years) with known or suspected IBDs undergoing IUD insertion between November 1st, 2019 and September 7th, 2022.

According to the clinic practice pattern (Figure S1), participants continued their prior hormonal therapy for at least 30 days after insertion. IUD insertion was within 60 days of the last depot medroxyprogesterone acetate injection to ensure adequate coverage for 30 days. Assessed data included the incidence of IUD expulsion, bleeding disorder diagnosis, mode of menstrual suppression in the first 30 days after insertion, subjective reported bleeding profiles at follow‐up, and any observed complications. Bleeding patterns were categorized as amenorrheic, light, normal, or heavy.^9^

## 3 RESULTS

Chart review identified 24 IUD insertions in 22 adolescents (Table 1). First follow‐up for all 24 insertions occurred approximately 30 days after insertion (mean 30 days, range 11–58 days). For patients initially seen at <30 days, a second follow‐up visit was reviewed to confirm at least 30‐day compliance with menstrual suppression, evaluate bleeding pattern, and assess IUD status. Hormonal menstrual suppression was utilized for 22 of 24 (92%) insertions. Menstrual suppression was shown to be effective, with 14 of 24 patients (58%) reporting light bleeding and seven of 24 patients (29%) reporting amenorrhea (Table). Altogether, no early expulsions because of excess bleeding were noted after any of the 24 IUD insertions. There were no expulsions because of heavy bleeding before 30 days for 21 of 22 individuals. One individual with type IIA von Willebrand disease had two expulsions (the first within 30 days of insertion and the second within 4 months [137 days]), neither associated with excessive bleeding (Table 1). Given the absence of HMB, expulsions in this youngest individual in the cohort were likely attributable to uterine size.^5^ A subsequent 19.5‐mg LNG‐IUD inserted 1 year later remained in place for >12 months. One individual experienced partial expulsion in the setting of excess bleeding more than 6 months after placement, supporting the relationship between heavy blood flow and displacement of the IUD from the endometrial cavity. One IUD was electively removed within 4 months because of cramping without excessive bleeding. No other expulsions were observed at the final follow‐up. This includes five individuals evaluated through 6 months and 11 individuals followed through 12 months.

**TABLE 1:** Participant demographics and clinical outcomes.

Diagnosis	Patients (*#*)	Age at insertion (years)	Menstrual suppression	First follow‐up		Final follow‐up		Expulsion	Time to expulsion (days)
				Time from insertion (days)	Bleeding pattern	Time from insertion (days)	Bleeding pattern		
HPS	1	18	POP	49	Light	326	Light	No	
EDS	2	19	DMPA	43	Light	1033	Light	No	
	3	18	POP	35	Light	63	Light	No	
	4	17	POP	30	Light	188	Heavy	Yes	188
	5	15	DMPA	31	Light	‐	‐	‐	‐
VWD type I	6	16	cOCP	13	Light	623	Light	No	
	7	16	DMPA	13	Amenorrheic	257	Amenorrheic	No	
	8	15	cOCP	26	Light	1003	Amenorrheic	No	
	9	15	cCOP	34	Light	891	Light	No	
	10	15	cOCP	12	Light	‐	‐	‐	
VWD type IIA^a^	11	11	cOCP	25	Light			Yes	120
		12	cOCP	29	Normal	137	Normal	Yes	137
		13	cOCP	58	Amenorrheic	252	Amenorrheic	No	
VWD type III	12	21	cOCP	53	Amenorrheic	1146	Amenorrheic	No	
		15	cOCP, TXA	11	Amenorrheic	649	Amenorrheic	No	
	13								
Factor VIII deficiency	14	15	cOCP	18	Amenorrheic	527	Light	No	
Factor IX deficiency	15	16	cOCP	16	Amenorrheic	825	Amenorrheic	No	
HMB (no diagnosis)	16	17	POP	37	Light	669	Amenorrheic	No	
	17	18	cOCP, TXA	20	Light	50	Amenorrheic	No	
	18	16	TXA	37	Light	897	Amenorrheic	No	
	19	16	Nexplanon	23	Amenorrheic	‐	‐	‐	
	20	16	cOCP	15	Amenorrheic	169	Amenorrheic	No	
	21	14	cOCP, TXA	35	Light	282	Amenorrheic	No	
	22	16	DMPA	41	Normal	446	Light	No	

*Note*: Participant age, diagnosis, use of menstrual suppressive agents, bleeding pattern at 4–6 weeks after insertion, and intrauterine device expulsion in patients with bleeding disorders.

Abbreviations: cOCP, combined oral contraceptive pill; DMPA, depot medroxyprogesterone acetate; EDS, Ehlers‐Danlos syndrome; HMB, heavy menstrual bleeding; HPS, Hermansky‐Pudlak syndrome; POP, progestin only pill; TXA, tranexamic acid; VWD, von Willebrand disease.

^a^ 19.5 mg levonorgestrel‐containing intrauterine device.

At final follow‐up, for those whose IUDs remained in place, bleeding was well controlled; 11 of 19 (58%) had amenorrhea and seven of 19 (36%) had light bleeding. In addition, there was no uterine perforation, hemorrhage, or bleeding that required further hemostatic interventions.

## 4 DISCUSSION

No early (≤30 days after insertion) IUD expulsions because of heavy bleeding were observed. If the risk for IUD expulsion in IBDs is decreased during the first menses following insertion, the LNG‐IUD's favorable bleeding profile may contribute to lower rates of late expulsion once patients experience a reduction in monthly blood loss.^2^

While acknowledging that our study is limited by loss of follow‐up and subjective assessment of bleeding profile improvement, the absence of early expulsion observed here in the context of menstrual suppression is a promising improvement over similarly sized studies that have reported higher rates of IUD expulsion in adolescents with IBDs.^2^, ^6^, ^7^

The LNG‐IUD is a safe and effective method of managing HMB in adolescents with IBDs. Concurrent menstrual suppression for at least 30 days after insertion, to prevent bleeding‐related early expulsion in this population, may maximize safety and minimize complications for this at‐risk group.

## AUTHOR CONTRIBUTIONS

Kelly M. Kons was involved in the design, chart review, data curation, formal analysis, and preparation of the manuscript. Megan H. Fiorillo was involved in study conceptualization, chart review, data curation, and preparation of the manuscript. Peter H. Cygan and Tonya S. Wright were involved in study conceptualization and design, data curation and analysis, project administration, manuscript preparation, and supervision. Peter H. Cygan and Tonya S. Wright are co‐corresponding authors.

## FUNDING INFORMATION

Peter H. Cygan receives research funding from EPBDF and NIH. He receives funding from HRSA and a clinical trial funded by Bloodworks Northwest.

## CONFLICT OF INTEREST STATEMENT

The authors declare no conflicts of interest.

## Supporting information


**Figure S1.** Risk‐adapted approach to intrauterine device placement in adolescents with bleeding disorders. Preoperative, perioperative/intraoperative, and postoperative considerations in patient‐specific clinical protocols to reduce bleeding and intrauterine device (IUD) expulsion in patients with bleeding disorders. Due to potential bleeding risk, nonsteroidal anti‐inflammatory drugs are generally avoided in this protocol. CBC, complete blood (cell) count; LNG, levonorgestrel; OCP, oral contraceptive pill; PBAC, pictorial blood loss assessment chart; TIBC, total iron‐binding capacity; UPT, urine pregnancy test. Figure adapted from Fiorillo et al., Obstetrics & Gynecology, 2022.

## Data Availability

Research data are not shared.
