# Supplementary material for: Menstrual suppression to decrease intrauterine device expulsion in adolescents with inherited bleeding disorders
Source: Int J Gynaecol Obstet. 2024 Nov 25;169(1):421–3. doi: 10.1002/ijgo.16063 (PMC11911950; doi:10.1002/ijgo.16063)
Supplement: Supplementary file 1 — Figure S1. Risk‐adapted approach to intrauterine device placement in adolescents with bleeding disorders. Preoperative, perioperative/intraoperative, and postoperative considerations in patient‐specific clinical protocols to reduce bleeding and intrauterine device (IUD) expulsion in patients with bleeding disorders. Due to potential bleeding risk, nonsteroidal anti‐inflammatory drugs are generally avoided in this protocol. CBC, complete blood (cell) count; LNG, levonorgestrel; OCP, oral contraceptive pill; PBAC, pictorial blood loss assessment chart; TIBC, total iron‐binding capacity; UPT, urine pregnancy test. Figure adapted from Fiorillo et al., Obstetrics & Gynecology, 2022. [file IJGO-169-421-s001.pptx]

## Slide 1
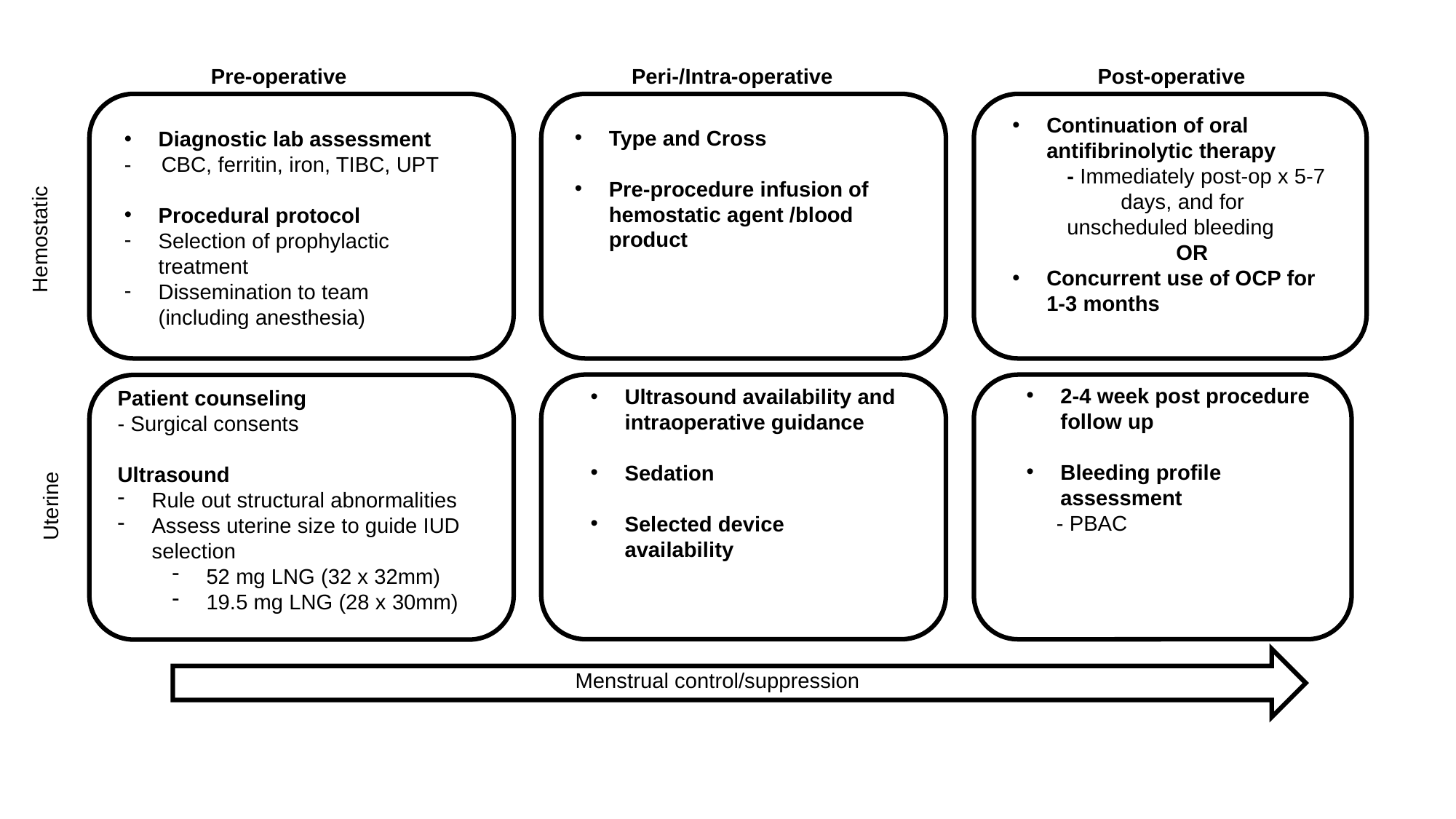

Pre-operative
Peri-/Intra-operative
Post-operative
Continuation of oral antifibrinolytic therapy
- Immediately post-op x 5-7 days, and for unscheduled bleeding
	OR
Concurrent use of OCP for 1-3 months
Type and Cross
Pre-procedure infusion of hemostatic agent /blood product
Diagnostic lab assessment
- CBC, ferritin, iron, TIBC, UPT
Procedural protocol
Selection of prophylactic treatment
Dissemination to team (including anesthesia)
Hemostatic
2-4 week post procedure follow up
Bleeding profile assessment
 - PBAC
Ultrasound availability and intraoperative guidance
Sedation
Selected device availability
Patient counseling
- Surgical consents
Ultrasound
Rule out structural abnormalities
Assess uterine size to guide IUD selection
52 mg LNG (32 x 32mm)
19.5 mg LNG (28 x 30mm)
Uterine
Menstrual control/suppression
